# Intravascular Optical-Resolution Photoacoustic Tomography with a 1.1 mm Diameter Catheter

**DOI:** 10.1371/journal.pone.0092463

**Published:** 2014-03-20

**Authors:** Xiaosong Bai, Xiaojing Gong, William Hau, Riqiang Lin, Jiaxiang Zheng, Chengbo Liu, Chengzhi Zeng, Xin Zou, Hairong Zheng, Liang Song

**Affiliations:** 1 Research Lab for Biomedical Optics and Molecular Imaging, Shenzhen Key Lab for Molecular Imaging, Institute of Biomedical and Health Engineering, Shenzhen Institutes of Advanced Technology, Chinese Academy of Sciences, Shenzhen, China; 2 Li Ka Shing Faculty of Medicine, The University of Hong Kong, Hong Kong, China; 3 Paul C. Lauterbur Research Center for Biomedical Imaging, Institute of Biomedical and Health Engineering, Shenzhen Institutes of Advanced Technology, Chinese Academy of Sciences, Shenzhen, China; 4 Beijing Center for Mathematics and Information Interdisciplinary Sciences (BCMIIS), Beijing, China; University of Minnesota, United States of America

## Abstract

Photoacoustic imaging is an emerging technology that can provide anatomic, functional, and molecular information about biological tissue. Intravascular spectroscopic and molecular photoacoustic imaging can potentially improve the identification of atherosclerotic plaque composition, the detection of inflammation, and ultimately the risk stratification of atherosclerosis. In this study, a first-of-its-kind intravascular optical-resolution photoacoustic tomography (OR-PAT) system with a 1.1 mm diameter catheter is developed, offering optical-diffraction limited transverse resolution as fine as 19.6 μm, ∼10-fold finer than that of conventional intravascular photoacoustic and ultrasonic imaging. To offer complementary imaging information and depth, the system also acquires co-registered intravascular ultrasound images in parallel. Imaging of an iliac stent and a lipid phantom shows that the high resolution and contrast of OR-PAT can enable improved stent implantation guidance and lipid identification. In the future, these capabilities may ultimately improve the diagnosis and interventional treatment of vulnerable atherosclerotic plaques, which are prone to cause thrombotic complications such as myocardial infarction and stroke.

## Introduction

Coronary artery disease is the leading cause of death in the developed world and is expected to rise in the coming years [1], [2]. Currently, coronary angiography is the gold standard for the assessment of coronary artery disease and for guiding percutaneous coronary intervention (PCI). However, it has significant limitations for assessing the extent and the severity of atherosclerosis, as it only allows a two-dimensional evaluation of the luminal dimensions and is unable to provide quantitative measurement of the atherosclerotic plaque burden and compositions [3]. The development of catheter-based intravascular imaging technologies, including intravascular ultrasound (IVUS) and intravascular optical coherence tomography (IV-OCT) [4], [5], has greatly enriched our knowledge of coronary atherosclerosis, and made it possible for the visualization, quantification, and characterization of atherosclerotic plaque burden, as well as the guidance of intervention.

In clinical medicine, the identification of the rupture-prone or the so-called vulnerable plaque is essential, as it is the precursor lesion for plaque rupture, and can potentially lead to the formation of thrombosis that is believed to be the main cause of sudden coronary death [6]. A vulnerable plaque typically consists of a lipid-rich necrotic core and a thin fibrous cap (≤ 65 μm), accompanied by inflammation and/or macrophage infiltration [7]. Unfortunately, existing mainstream intravascular imaging technologies, including IVUS and IV-OCT, still have certain limitations in accurately assessing these characteristics for identifying vulnerable plaques. For IVUS, it is capable of visualizing the comprehensive structure of the arterial wall, but its spatial resolution and/or contrast are insufficient to resolve the microscopic structure of atherosclerotic plaques, such as the thin fibrous cap. For IV-OCT, it can provide unique insights into certain microscopic features of vulnerable plaques with high spatial resolution, but its penetration depth is limited to only ∼1 mm. Further, neither IVUS nor IV-OCT can accurately identify the molecular composition and inflammation activities of atherosclerotic plaques, both of which are critical factors closely related to the vulnerability of the plaques.

Photoacoustic imaging is a rapidly emerging technology capable of providing anatomic, functional, and molecular information about biological tissue [8]-[14]. In photoacoustic imaging, a nanosecond pulsed laser is usually employed to illuminate biological tissue; upon the illumination, acoustic (photoacoustic) waves will be induced due to a transient thermoelastic expansion of the tissue from the pulsed optical energy deposition. The photoacoustic waves are then collected by an ultrasonic transducer array (or a single-element ultrasonic transducer with scanning) to reconstruct a cross-section/tomographic photoacoustic image, reflecting essentially the spatial distribution of the optical absorption properties of the tissue. Since the optical absorption spectrum is a distinct signature of the molecular conformation and/or composition of tissue, multi-wavelength illumination can be employed for spectroscopic photoacoustic imaging to directly record the spectral information of the imaged biological tissue with extremely high sensitivity. An appropriate signal-processing algorithm can then be used to un-mix the recorded spectra, and ultimately to resolve the constituents of the imaged biological tissue. Hence, intravascular spectroscopic photoacoustic imaging inherently possesses an excellent capability to identify the atherosclerotic plaque composition, as already demonstrated by a number of previous studies [15]–[20].

During the past few years, a variety of intravascular photoacoustic imaging (IVPA) systems have been developed. In the first-generation system, the illumination laser beam for photoacoustic excitation was delivered from the outside of the blood vessel, while a catheter housing a high-frequency ultrasonic transducer was inserted into the blood vessel for photoacoustic detection [21]. Due to the external photoacoustic excitation, such a design was not suitable for intravascular imaging applications in reality. However, initial experiments using this design demonstrated the potential of IVPA for identifying different constituents of atherosclerotic plaques [21]. Later on, to enable truly intravascular photoacoustic imaging, a number of different catheter designs were proposed and implemented [22]–[24]. In general, such a system integrated a multimodal optical fiber, a light reflection component, and a miniature high-frequency ultrasonic transducer into a single catheter to allow both photoacoustic excitation and detection inside the vessel to be imaged; in the catheter, the optics was arranged either in a sequential or parallel manner with respect to the ultrasonics. In one study, an IVPA system with a 1.25 mm diameter catheter was developed to image excised human coronary segments (post-mortem) [25], [26], demonstrating the feasibility of identifying lipid contents in human atherosclerotic plaques using spectroscopic IVPA. Recently, with a 2.2 mm diameter catheter, IVPA was also successfully applied to image the aorta of a rabbit *in vivo*, further demonstrating the prospect of translating IVPA from the bench to the clinic [27], [28].

In this paper, we present a first-of-its-kind intravascular optical-resolution photoacoustic tomography (OR-PAT) system that can perform both anatomic and spectroscopic photoacoustic imaging at a microscopic level-the transverse resolution is as fine as 19.6 μm, close to that of OCT and ∼10 times better than that of conventional intravascular photoacoustic and ultrasonic imaging. The developed OR-PAT catheter has only an outer diameter of 1.1 mm, making it highly translatable for *in vivo* preclinical and clinical intravascular applications. To offer complementary imaging information and depth, the OR-PAT system also acquires co-registered intravascular ultrasound images in parallel. Imaging of an iliac stent and a lipid phantom shows that the high resolution and contrast of OR-PAT can potentially enable improved stent implantation guidance and lipid identification.

## Methods

### System architecture

**Figure 1:**
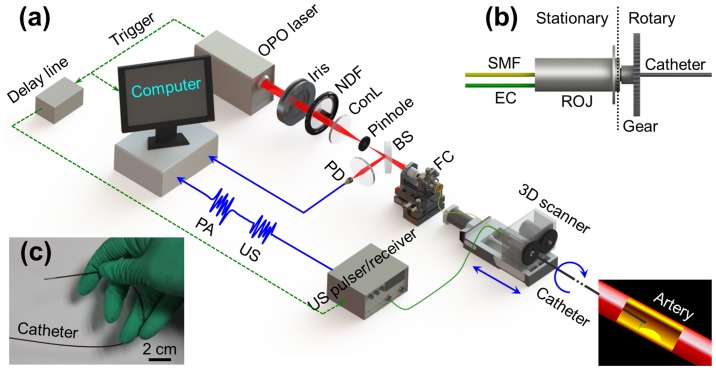
Illustration of the OR-PAT system. (a) Overall architecture of the system. (b) Schematic of the rotational mechanism of the catheter. (c) A photo of the catheter showing its flexibility. OPO, optical parametric oscillator; NDF, neutral density filter; ConL, condenser lens; BS: beam splitter; PD, photodiode; FC, fiber coupler; US, ultrasonics; PA, photoacoustics; SMF, single mode fiber; EC, electrical cable; 3D scanner, consisting of an optical-electric rotary joint (ROJ), a step motor, and a motorized pull-back stage.

Figure 1(a) illustrates the overall architecture of the OR-PAT system, which can simultaneously perform intravascular photoacoustic and ultrasonic imaging. For photoacoustic imaging, a pulsed OPO (optical parametric oscillator) laser (Vibrant B/355-II, OPOTEK) of a 10-Hz repetition rate was used, operating at a tunable near-infrared (NIR) wavelength around 1200 nm. The output laser beam (pulse width: 5 ns) was reshaped by an iris of 6 mm in diameter, attenuated by a neutral density filter, and then focused by a condenser lens before passing through a 50-μm pinhole for spatial filtering. The filtered beam was coupled into a single mode fiber (SMF) (Nufern, 1060XP) via an assembled fiber coupler. A beam sampler and a photodiode were used to monitor the fluctuation of laser energy per pulse. The output end of the SMF was connected to the stationary part of a fiber optic rotary joint (Princetel Inc., Pennington, New Jersey), coupling the laser beam into another SMF connected to the rotary part of the joint and further transmitting it to the distal end of the catheter to excite photoacoustic signals. The final output laser energy from the catheter tip was maintained to be ∼500 nJ/pulse. Following the widely used method to estimate the surface laser fluence in optical-resolution photoacoustic imaging [12], [14], we assume the laser was focused at ∼1 mm below the tissue surface; this energy corresponds to an optical fluence of only ∼7 mJ/cm^2^ per pulse at the tissue surface, below the 20-mJ/cm^2^ ANSI safety limit for skin surface at this wavelength. A custom-made electric slip ring, which also includes a stationary and a rotary part, was assembled co-axially with the fiber optic rotary joint to form an optical-electric rotary joint (Fig. 1(b)) that can rotate as fast as 1800 revolutions per minute (RPM). In addition, the flexibility required for catheterization was also taken into account while fabricating the catheter (Fig. 1(c)). Further details of the imaging catheter are given in section 2.2. To form an A-line (depth-encoded one-dimensional image), the photoacoustic waves emitted from the sample were detected by a custom-made miniature single-element ultrasonic transducer, placed at the tip of the catheter. Then, the output electrical signals from the transducer were amplified by an ultrasonic pulser-receiver, digitized via a 100-MS/s data acquisition (DAQ) card, and finally stored into a personal computer (PC). Cross-sectional image (B-scan) data were recorded by rotating the catheter with respect to its central axis via a step motor, which was synchronized with the pulsed laser firing. Then, the acquired (photoacoustic or ultrasonic) signals were band-pass filtered (bandwidth: 20–50 MHz), followed by Hilbert transform, and then converted to the polar coordinates for display (signal processing performed with MATLAB®). Three dimensional (3D) images, consisting of a series of B-scans, were recorded step-by-step by longitudinally translating the catheter via a step-motor driven pull-back stage. All the processes mentioned above were controlled by a custom-developed LabView program in the PC.

The intravascular ultrasound images were acquired in parallel to the photoacoustic imaging process described above. The synchronization signal from the OPO laser was delayed 30 μs by a delay line to allow the completion of photoacoustic recording before triggering the ultrasonic pulser-receiver for ultrasonic firing and receiving.

### Imaging catheter

**Figure 2:**
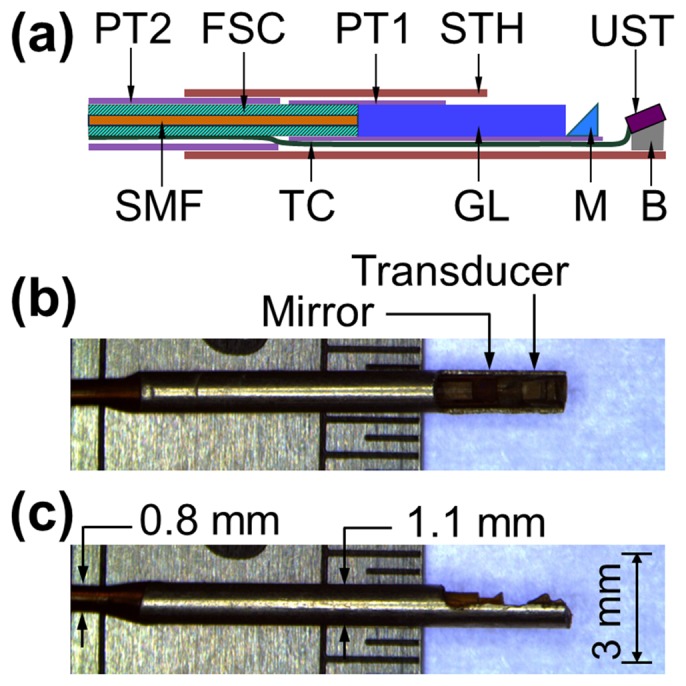
The distal end of the OR-PAT catheter. (a) Overall architecture. (b) Side and (c) Top view of the catheter under a conventional optical microscope. FSC, flexible stainless steel coil; PT1, polyimide tube 1 with an inner diameter of 0.7 mm; PT2, polyimide tube 2 with an inner diameter of 0.74 mm; STH, stainless steel tube housing; UST, ultrasonic transducer; SMF, single-mode fiber; TC, transducer cable; GL, gradient-index lens; M, micro-prism; B, metal base.

Figure 2(a) is a schematic of the distal end of the miniature OR-PAT catheter, consisting of multiple optical and acoustic components assembled in a stainless steel tube housing. The outer diameter of the catheter (housing) was 1.1 mm, comparable to the size of IVUS catheters used in the clinic (e.g., the Eagle Eye® Platinum catheter from Volcano Corporation), making it highly translatable for *in vivo* intravascular imaging. The SMF was capsuled by a 0.5-mm outer diameter flexible stainless steel coil to ensure effective transmission of rotational torque from the rotary joint to the probe distal end. A custom-designed gradient-index (GRIN) lens with a diameter of 0.5 mm and a working distance of 3 mm in air was used to focus the laser from the SMF tip to a 0.5-mm micro-prism, which then reflected the focused laser beam to the inner surface of the vessel wall. The distal end of the flexible stainless steel tube, the GRIN lens, and the micro-prism were fixed and sealed in a thin-wall polyimide tube (PT1) with an inner diameter of 0.7 mm and a wall thickness of 25 μm (Sanz Technologies, Beijing, China). An aperture was opened at the tip of the tube to allow the delivery of light for photoacoustic excitation. A custom-made ultrasonic transducer (Blatek, State College, PA) with a dimension of 0.6 mm × 0.5 mm × 0.2 mm (length × width × thickness), a center frequency of 40 MHz, and a fractional bandwidth of ∼60% was sequentially aligned with the optical components and mounted on a metal base behind the micro-prism. Note that the use of such an ultrasonic transducer has resulted in an acoustic near-field range of ∼2 mm, which theoretically should be avoided as much as possible. However, similar to that in the clinically used intravascular ultrasound, due to the very small size and high frequency required for such applications, it is very challenging to completely eliminate this issue. Fortunately, in most cases, although a small part of the target (stent/arterial wall) may be slightly in the near field, the acquired images are in general of a well acceptable quality to use. The center distance between the ultrasonic transducer and the micro-prism was finely adjusted to be ∼1 mm. The ultrasonic transducer is purposely tilted with an angle of 20 degree to increase the detection sensitivity to the excited photoacoustic waves. Finally, the rest of the flexible stainless steel coil out of PT1 and the ultrasonic transducer cable were sealed in another thin-wall polyimide tube (PT2) with an inner diameter of 0.74 mm and a wall thickness of 30 μm (Sanz Technologies, Beijing, China). As mentioned in the beginning of this section, all the optical and ultrasonic components at the distal end of the catheter were ultimately housed in the stainless steel tube housing with an outer diameter of 1.1 mm and an inner diameter of 0.9 mm, with an aperture opened at its tip to allow the free traveling of both light and ultrasound.

## Results

### Resolution measurement

**Figure 3:**
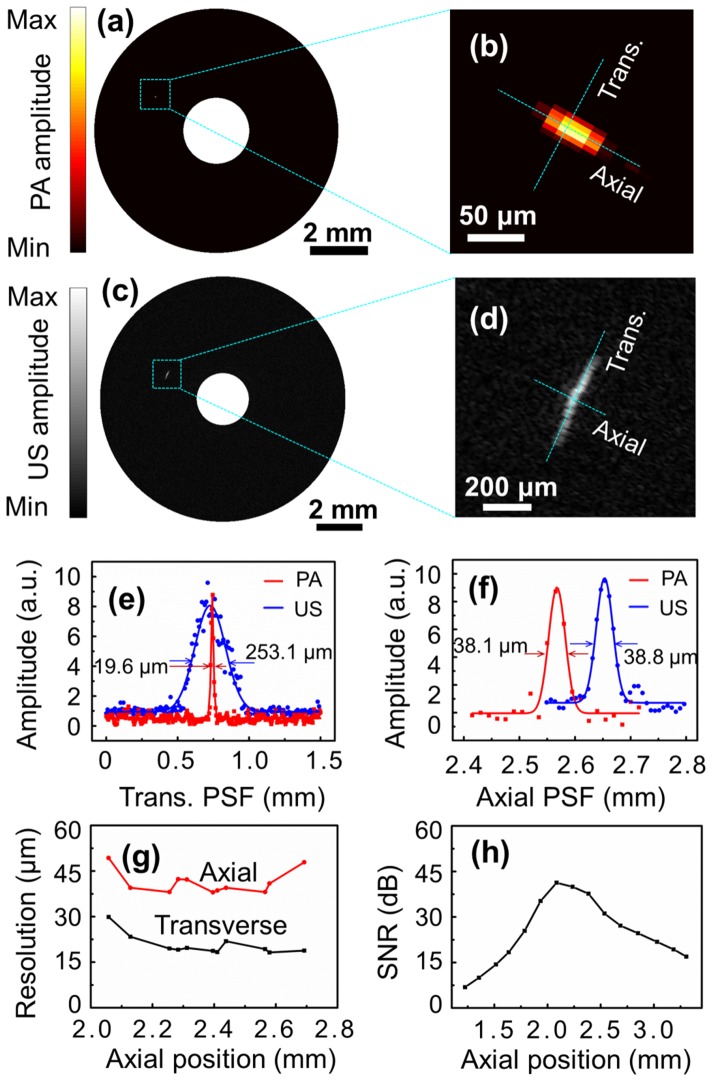
The spatial resolutions of photoacoustic and ultrasonic imaging of OR-PAT. (a) Photoacoustic B-scan image of a carbon fiber (6 μm in diameter). (b) Enlarged view of the dashed box in (a). (c) Ultrasonic B-scan image of a tungsten wire (12 μm in diameter). (d) Enlarged view of the dashed box in (c). (e) The transverse and (f) axial resolutions of photoacoustic (red) and ultrasonic (blue) imaging with OR-PAT. (g) The spatial resolutions and (h) SNR as a function of the axial position. PA, photoacoustics; US, ultrasonics; Trans., transverse; PSF, point spread function.

To measure the photoacoustic and ultrasonic resolutions of the OR-PAT system, a carbon fiber of 6 μm in diameter and a tungsten wire of 12 μm in diameter were photoacoustically and ultrasonically imaged, respectively. Figure 3(a) shows a photoacoustic B-scan image of the carbon fiber at an axial position of 2.6 mm, while Fig. 3(b) is an enlarged view of the dashed box in Fig. 3(a). Likewise, Fig. 3(c) shows an ultrasonic B-scan image of the tungsten wire at approximately the same axial position, while Fig. 3(d) is an enlarged view of the dashed box in Fig. 3(c). In Figs. 3(e) and 3(f), the dots represent the experimental data corresponding to the dotted lines in Figs. 3(b)and 3(d) along the transverse and axial directions, respectively. Further, the data is fitted with Gaussian functions to estimate the spatial resolutions in both directions. It can be seen that, in the axial direction, the two imaging modalities have similar spatial resolutions (∼38 μm), as determined primarily by the ultrasonic detection bandwidth; however, in the transverse direction, photoacoustic imaging offers a superior optical-diffraction limited spatial resolution of ∼19.6 μm, which is more than 10 times better than the 253.1 μm ultrasonic resolution (Fig. 3(e)). This transverse photoacoustic resolution of OR-PAT is also ∼10 times better than that of conventional IVUS technology and previously reported intravascular photoacoustic imaging systems [24]. In Fig. 3(g), the measured axial and transverse resolutions of photoacoustic imaging are plotted as a function of the axial positions. In Fig. 3(h), the signal-to-noise ratio (SNR) of photoacoustic imaging versus the axial position (at an interval of 150 μm) is plotted, using a black tape as the imaging target (laser energy ∼500 nJ/pulse).

### Imaging of an iliac stent

**Figure 4:**
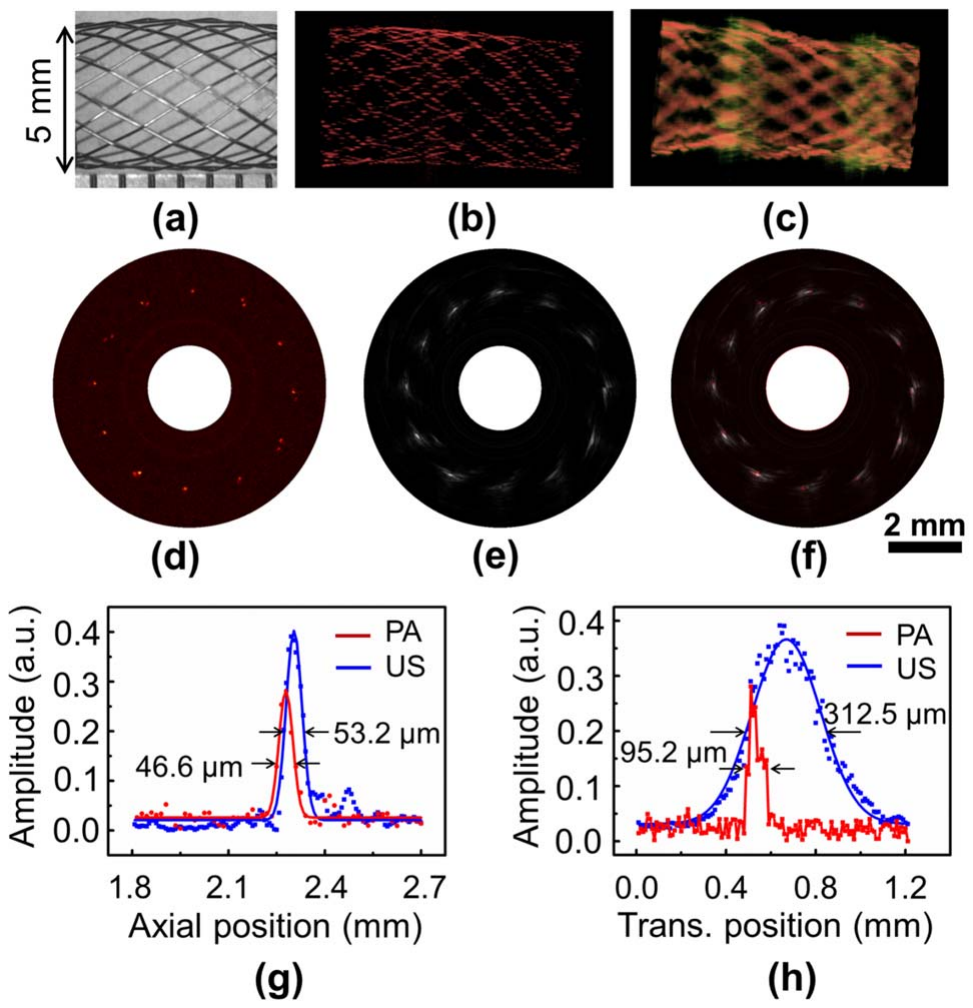
OR-PAT of an iliac/common femoral artery stent. (a) Optical microscopic image of the imaged sent segment. (b) Representative 3D photoacoustic and (c) ultrasonic images. (d) Photoacoustic, (e) ultrasonic, and (f) fused B-scan images of a cross section of the stent. (g) Axial and (h) transverse photoacoustic (red) and ultrasonic (blue) signal profiles of a wire junction at 2 o′clock in (f). PA, photoacoustics; US, ultrasonics; Trans., transverse.

To demonstrate the 3D intravascular imaging capability of OR-PAT, an iliac/common femoral artery stent (Boston Scientific Corporation) consisting of 24 individual metallic struts (each of a diameter of 70 μm) was dilated to a diameter of 5 mm and imaged in water by inserting the OR-PAT catheter into the center of the stent. For each A-line, photoacoustic and ultrasonic signals were recorded sequentially with a delay of 30 μs in between, within which the rotation of the catheter was negligible. Therefore, during 2D and 3D imaging, photoacoustic and ultrasonic images were acquired essentially simultaneously. While Fig. 4(a) is a digital photograph of the imaged stent segment under optical microscope, Figs. 4(b) and 4(c) are two representative 3D photoacoustic and ultrasonic images featuring the strut mesh of the stent. Typical photoacoustic and ultrasonic B-scan images showing the cross section of the stent are also shown (Figs. 4(d) and 4(e)). It can be seen that, both modalities can image the metallic stent with high contrast; however, compared with ultrasonic imaging, optical-resolution photoacoustics is shown to be able to visualize finer features of the stent at a much higher resolution. Further, the images from these two modalities, simultaneously acquired with our OR-PAT system, co-register well with each other (Fig. 4(f)). To compare the photoacoustic and ultrasonic resolutions in this particular case, the cross-section profiles of a strut junction at 2 o′clock (Fig. 4(f)) along the transverse and axial directions are plotted in Figs. 4(g) and 4(h).

**Figure 5:**
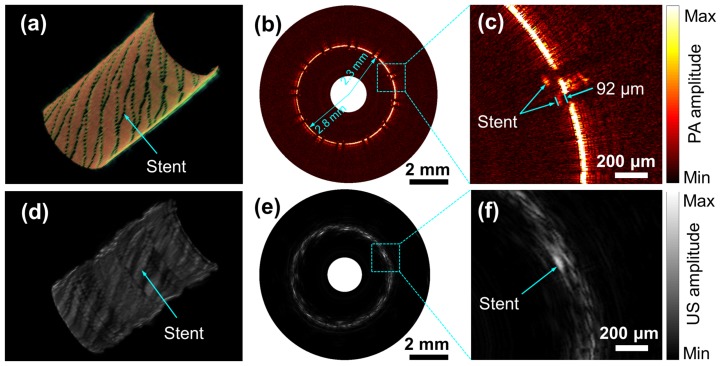
Photoacoustic and ultrasonic imaging with OR-PAT of a stent deployed in a plastic tube. Three-dimensional cut-away (a) photoacoustic and (d) ultrasonic images. Representative (b) photoacoustic and (e) ultrasonic B-scans. Enlarged photoacoustic (c) and ultrasonic images corresponding to the dash boxes in (b) and (e).

To further demonstrate the potential application of OR-PAT for guiding and assessing stent implantation, the iliac stent was carefully deployed into a plastic tube mimicking a vessel. The 3D cut-away photoacoustic and ultrasonic images of the deployed stent are shown in Figs. 5(a) and 5(d). Figures 5(b) and 5(e) are representative photoacoustic and ultrasonic B-scans from Figs. 5(a) and 5(d), respectively. In Figs. 5(c) and 5(f), two small areas including both the stent strut and the tube wall are enlarged to show the apposition of the stent. It can be seen that, with optical-resolution photoacoustic imaging, even a tiny gap smaller than 0.1 mm between the stent and the tube wall can be clearly identified; while with ultrasonic imaging, this gap is not well resolved. In photoacoustic imaging, due to the high resolution and strong attenuation of the excitation light by the stent, shadows of the stent struts similar to those in IV-OCT images are observed (Fig. 5(b)).

### Imaging of lipid content

**Figure 6:**
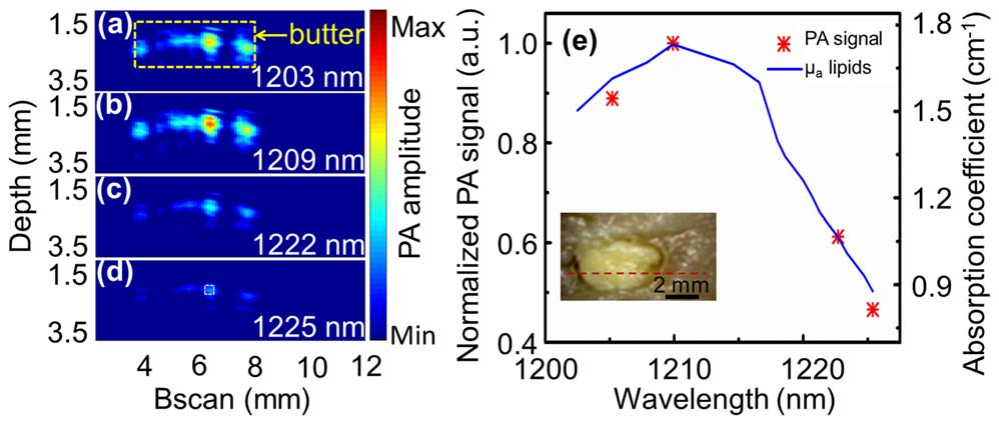
Spectroscopic OR-PAT of lipid. (a)–(d) Photoacoustic images at four different laser wavelengths of a phantom made by placing a small amount of butter into chicken breast tissue (see inset in (e)). The yellow box in (a) corresponds to the butter content in (e), while the white box in (d) indicates the area that photoacoustic signals are averaged for the photoacoustic spectra plot in (e). (e) Comparison between the acquired photoacoustic spectra of butter and previously reported optical absorption spectra of lipid [29].

To demonstrate the feasibility of identifying plaque composition with OR-PAT, a physical phantom fabricated by placing a small amount of butter into a chicken breast chunk was photoacoustically imaged. The photograph of the phantom is shown as an inset in Fig. 6(e). Four wavelengths, at 1203, 1209, 1222, and 1225 nm, respectively, were used to perform spectroscopic photoacoustic imaging. To increase the SNR, the images were averaged 30 times for each A-line. In addition, to avoid excessively long imaging time using the 10-Hz repetition rate laser, the experiments were performed by linearly translating the phantom at a relatively large step size of 100 μm. The acquired photoacoustic B-scans corresponding to the dash red line in the inset of Fig. 6(e) are shown in Figs. 6(a)–6(d). Further, the photoacoustic signals corresponding to the white dash box in Fig. 6(d) are averaged and plotted as a function of excitation wavelength (Fig 6(e)). It can be seen that, the experimentally measured photoacoustic spectra of lipid (butter) agrees very well with the absorption spectra of lipid reported in the literature [29], confirming the potential of using OR-PAT to image and identify lipid content in atherosclerotic plaques.

## Discussion and Conclusions

In this study, an intravascular optical-resolution photoacoustic tomography (OR-PAT) system that can simultaneously perform photoacoustic and ultrasonic imaging is developed. The system's imaging catheter has an outer diameter as small as 1.1 mm and offers optical-diffraction limited transverse photoacoustic resolution of 19.6 μm, ∼10-fold better than that of conventional intravascular photoacoustic and ultrasonic imaging. To the best of our knowledge, this is the first report of a catheter-based photoacoustic imaging system that can provide optical-diffraction limited spatial resolution. The OR-PAT system is developed upon overcoming a number of challenges, including: (1) the design of light delivery and focus in a ∼1-mm scale for effective photoacoustic excitation, with a laser energy as low as ∼500 nJ/pulse; (2) the design of a catheter architecture capable of detecting the excited photoacoustic waves at high efficiency; and (3) the effective transmission of both pulsed tunable laser and high-frequency electrical signals to and/or from a rotating catheter for robust 3D imaging.

To demonstrate the potential clinical applications of OR-PAT, an iliac stent deployed in a vessel phantom has been imaged, exhibiting excellent contrast and resolution that can potentially improve stent implantation guidance. In addition, using spectroscopic OR-PAT, lipid content has been successfully identified. With its built-in intravascular ultrasonic imaging capability, OR-PAT offers not only complementary structural and functional information, but also complementary spatial resolution and penetration depth: while the optical-resolution photoacoustic imaging measures the microscopic functional/compositional characteristics of the atherosclerotic plaques, the ultrasonic imaging can penetrate the entire vessel wall to evaluate the overall plaque burden. As a result, OR-PAT can potentially increase the accuracy for assessing the severity and vulnerability of atherosclerotic plaques and enable the development of improved interventional treatment for atherosclerosis. Finally, because a focused laser beam is used for photoacoustic excitation, the required laser energy per pulse for intravascular photoacoustic imaging has been reduced by >1000 folds compared with previously reported studies (from a few mJ to ∼500 nJ). Consequently, unlike previous IVPA systems that require a multimode optical fiber for light delivery, a single-mode optical fiber can be used in OR-PAT, which, in turn, opens up new opportunities to: (1) reduce the diameter and increase the flexibility of the catheter; (2) further integrate IV-OCT into the system for high-resolution structural imaging.

For future studies, in order to enable broader potential intravascular imaging applications, the OR-PAT catheter needs to be further downsized to a diameter of <1 mm, and the length of the rigid part at the catheter's distal end should also be further reduced (for example, by using a ball lens instead of a GRIN lens to focus the laser beam). In addition, similar to previously reported IVPA systems, the current imaging speed of OR-PAT is also limited by the slow (10-Hz) repetition rate of the pulsed tunable laser, which has long been a challenge for translating intravascular photoacoustics towards *in vivo* imaging. In particular, because the number of A-lines has increased to 1600 lines/B-scan (∼5-fold more compared with that of conventional IVPA) in order to maintain the high lateral resolution, the imaging speed of the current OR-PAT system is relatively low (160 s/B-scan). Fortunately, because OR-PAT uses 1000-fold less optical energy per pulse for photoacoustic excitation, it has a great potential to break through this imaging speed limitation, given that the latest high-repetition-rate OPO laser can already offer a repetition rate of up to 1–2 kHz and an output energy of tens of μJ/pulse. In the future, by taking the privilege of using such high-repetition-rate OPO laser sources, OR-PAT may open up new opportunities to significantly (10–20 folds) improve the imaging speed of intravascular photoacoustic imaging.
